# Supplementary material for: Heparin-based hydrogel scaffolding alters the transcriptomic profile and increases the chemoresistance of MDA-MB-231 triple-negative breast cancer cells
Source: Biomater Sci. 2020 Feb 13;8(10):2786–96. doi: 10.1039/c9bm01481k (PMC7497406; doi:10.1039/c9bm01481k)
Supplement: Supplementary file 2 [file BM-008-C9BM01481K-s002.zip › Supplementary File 4/EGFvControl/Pathways/my_analysis.Gsea.1545200981068/HALLMARK_FATTY_ACID_METABOLISM.html]

Details for gene set HALLMARK\_FATTY\_ACID\_METABOLISM[GSEA]

|  || Dataset | expr.class.cls#EGF\_versus\_CONTROL.class.cls#EGF\_versus\_CONTROL\_repos |
| Phenotype | class.cls#EGF\_versus\_CONTROL\_repos |
| Upregulated in class | EGF |
| GeneSet | HALLMARK\_FATTY\_ACID\_METABOLISM |
| Enrichment Score (ES) | 0.24571249 |
| Normalized Enrichment Score (NES) | 1.080625 |
| Nominal p-value | 0.30387598 |
| FDR q-value | 0.38591966 |
| FWER p-Value | 1.0 |
Table: GSEA Results Summary

  

Fig 1: Enrichment plot: HALLMARK\_FATTY\_ACID\_METABOLISM      
 Profile of the Running ES Score & Positions of GeneSet Members on the Rank Ordered List

  

| PROBE | DESCRIPTION (from dataset) | GENE SYMBOL | GENE\_TITLE | RANK IN GENE LIST | RANK METRIC SCORE | RUNNING ES | CORE ENRICHMENT || 1 | CYP1A1 | na |  |  | 96 | 2.351 | 0.0205 | Yes |
| 2 | AOC3 | na |  |  | 291 | 1.966 | 0.0317 | Yes |
| 3 | ODC1 | na |  |  | 415 | 1.835 | 0.0452 | Yes |
| 4 | FH | na |  |  | 638 | 1.677 | 0.0518 | Yes |
| 5 | SLC22A5 | na |  |  | 839 | 1.573 | 0.0584 | Yes |
| 6 | AADAT | na |  |  | 845 | 1.570 | 0.0752 | Yes |
| 7 | GCDH | na |  |  | 884 | 1.559 | 0.0902 | Yes |
| 8 | NCAPH2 | na |  |  | 1056 | 1.491 | 0.0974 | Yes |
| 9 | CBR1 | na |  |  | 1267 | 1.424 | 0.1019 | Yes |
| 10 | MDH1 | na |  |  | 1318 | 1.406 | 0.1145 | Yes |
| 11 | ME1 | na |  |  | 1379 | 1.385 | 0.1264 | Yes |
| 12 | SMS | na |  |  | 1405 | 1.376 | 0.1401 | Yes |
| 13 | NBN | na |  |  | 1465 | 1.358 | 0.1518 | Yes |
| 14 | PTS | na |  |  | 1498 | 1.346 | 0.1647 | Yes |
| 15 | ACSL4 | na |  |  | 1621 | 1.312 | 0.1726 | Yes |
| 16 | SUCLA2 | na |  |  | 1863 | 1.254 | 0.1736 | Yes |
| 17 | ELOVL5 | na |  |  | 1901 | 1.246 | 0.1852 | Yes |
| 18 | RAP1GDS1 | na |  |  | 2107 | 1.195 | 0.1874 | Yes |
| 19 | DHCR24 | na |  |  | 2137 | 1.190 | 0.1988 | Yes |
| 20 | HSPH1 | na |  |  | 2205 | 1.177 | 0.2081 | Yes |
| 21 | ECI2 | na |  |  | 2215 | 1.174 | 0.2204 | Yes |
| 22 | PDHA1 | na |  |  | 2270 | 1.162 | 0.2302 | Yes |
| 23 | METAP1 | na |  |  | 2543 | 1.103 | 0.2279 | Yes |
| 24 | ACAT2 | na |  |  | 3122 | 1.002 | 0.2085 | Yes |
| 25 | PCBD1 | na |  |  | 3433 | 0.948 | 0.2025 | Yes |
| 26 | HSD17B10 | na |  |  | 3686 | 0.907 | 0.1992 | Yes |
| 27 | YWHAH | na |  |  | 3864 | 0.875 | 0.1994 | Yes |
| 28 | DLD | na |  |  | 3868 | 0.873 | 0.2087 | Yes |
| 29 | UROS | na |  |  | 3873 | 0.873 | 0.2180 | Yes |
| 30 | ADIPOR2 | na |  |  | 3953 | 0.860 | 0.2232 | Yes |
| 31 | H2AFZ | na |  |  | 4017 | 0.848 | 0.2291 | Yes |
| 32 | SDHD | na |  |  | 4053 | 0.843 | 0.2365 | Yes |
| 33 | GPD2 | na |  |  | 4376 | 0.795 | 0.2282 | Yes |
| 34 | CPT2 | na |  |  | 4647 | 0.756 | 0.2223 | Yes |
| 35 | ADSL | na |  |  | 4784 | 0.733 | 0.2231 | Yes |
| 36 | NSDHL | na |  |  | 4974 | 0.712 | 0.2210 | Yes |
| 37 | OSTC | na |  |  | 5012 | 0.706 | 0.2267 | Yes |
| 38 | CRYZ | na |  |  | 5019 | 0.705 | 0.2340 | Yes |
| 39 | ACAA1 | na |  |  | 5086 | 0.695 | 0.2381 | Yes |
| 40 | ACSL1 | na |  |  | 5124 | 0.689 | 0.2437 | Yes |
| 41 | HIBCH | na |  |  | 5226 | 0.675 | 0.2457 | Yes |
| 42 | SDHA | na |  |  | 5594 | 0.627 | 0.2333 | No |
| 43 | LTC4S | na |  |  | 5632 | 0.620 | 0.2381 | No |
| 44 | ALDH9A1 | na |  |  | 5781 | 0.599 | 0.2368 | No |
| 45 | EHHADH | na |  |  | 5911 | 0.581 | 0.2364 | No |
| 46 | SUCLG2 | na |  |  | 5942 | 0.578 | 0.2411 | No |
| 47 | HSDL2 | na |  |  | 6081 | 0.560 | 0.2399 | No |
| 48 | MGLL | na |  |  | 6147 | 0.549 | 0.2425 | No |
| 49 | ACOT2 | na |  |  | 6286 | 0.529 | 0.2410 | No |
| 50 | ACADM | na |  |  | 6509 | 0.502 | 0.2348 | No |
| 51 | ECI1 | na |  |  | 6530 | 0.498 | 0.2392 | No |
| 52 | CCDC58 | na |  |  | 7019 | 0.444 | 0.2184 | No |
| 53 | BMPR1B | na |  |  | 7163 | 0.426 | 0.2156 | No |
| 54 | MLYCD | na |  |  | 7518 | 0.383 | 0.2012 | No |
| 55 | ERP29 | na |  |  | 7527 | 0.382 | 0.2049 | No |
| 56 | IDH3B | na |  |  | 7605 | 0.373 | 0.2049 | No |
| 57 | HCCS | na |  |  | 7731 | 0.359 | 0.2022 | No |
| 58 | UROD | na |  |  | 7917 | 0.336 | 0.1962 | No |
| 59 | DLST | na |  |  | 8145 | 0.311 | 0.1877 | No |
| 60 | BCKDHB | na |  |  | 8201 | 0.305 | 0.1881 | No |
| 61 | MAOA | na |  |  | 8409 | 0.283 | 0.1803 | No |
| 62 | SUCLG1 | na |  |  | 8421 | 0.282 | 0.1828 | No |
| 63 | HMGCS1 | na |  |  | 8453 | 0.278 | 0.1842 | No |
| 64 | PRDX6 | na |  |  | 8480 | 0.274 | 0.1858 | No |
| 65 | ACADL | na |  |  | 8492 | 0.273 | 0.1882 | No |
| 66 | IDH1 | na |  |  | 8504 | 0.271 | 0.1906 | No |
| 67 | D2HGDH | na |  |  | 8536 | 0.267 | 0.1919 | No |
| 68 | DECR1 | na |  |  | 8591 | 0.263 | 0.1919 | No |
| 69 | SDHC | na |  |  | 8704 | 0.246 | 0.1887 | No |
| 70 | ACAA2 | na |  |  | 8723 | 0.243 | 0.1904 | No |
| 71 | ACOX1 | na |  |  | 9429 | 0.166 | 0.1552 | No |
| 72 | HADH | na |  |  | 9445 | 0.164 | 0.1562 | No |
| 73 | CPT1A | na |  |  | 9521 | 0.154 | 0.1540 | No |
| 74 | PDHB | na |  |  | 9808 | 0.125 | 0.1403 | No |
| 75 | IDI1 | na |  |  | 9872 | 0.119 | 0.1383 | No |
| 76 | HSD17B11 | na |  |  | 10030 | 0.103 | 0.1312 | No |
| 77 | PTPRG | na |  |  | 10288 | 0.071 | 0.1185 | No |
| 78 | MDH2 | na |  |  | 10374 | 0.061 | 0.1147 | No |
| 79 | ENO3 | na |  |  | 10445 | 0.059 | 0.1117 | No |
| 80 | RDH11 | na |  |  | 10948 | 0.003 | 0.0854 | No |
| 81 | ECHS1 | na |  |  | 11160 | -0.022 | 0.0745 | No |
| 82 | HADHB | na |  |  | 11243 | -0.033 | 0.0706 | No |
| 83 | ACSM3 | na |  |  | 11296 | -0.039 | 0.0683 | No |
| 84 | CPOX | na |  |  | 11388 | -0.048 | 0.0640 | No |
| 85 | CBR3 | na |  |  | 11573 | -0.066 | 0.0551 | No |
| 86 | MCEE | na |  |  | 11751 | -0.092 | 0.0468 | No |
| 87 | APEX1 | na |  |  | 11959 | -0.120 | 0.0373 | No |
| 88 | AUH | na |  |  | 12226 | -0.146 | 0.0249 | No |
| 89 | RETSAT | na |  |  | 12351 | -0.162 | 0.0202 | No |
| 90 | G0S2 | na |  |  | 12437 | -0.176 | 0.0177 | No |
| 91 | AQP7 | na |  |  | 12537 | -0.189 | 0.0145 | No |
| 92 | UGDH | na |  |  | 13035 | -0.249 | -0.0088 | No |
| 93 | FASN | na |  |  | 13229 | -0.278 | -0.0159 | No |
| 94 | HSD17B7 | na |  |  | 13436 | -0.306 | -0.0234 | No |
| 95 | ACSS1 | na |  |  | 13528 | -0.321 | -0.0247 | No |
| 96 | PPARA | na |  |  | 13529 | -0.321 | -0.0212 | No |
| 97 | ACO2 | na |  |  | 13859 | -0.360 | -0.0346 | No |
| 98 | ETFDH | na |  |  | 14057 | -0.387 | -0.0407 | No |
| 99 | GPD1 | na |  |  | 14117 | -0.394 | -0.0395 | No |
| 100 | ALDH3A2 | na |  |  | 14178 | -0.402 | -0.0383 | No |
| 101 | BLVRA | na |  |  | 14181 | -0.403 | -0.0340 | No |
| 102 | IDH3G | na |  |  | 14200 | -0.405 | -0.0305 | No |
| 103 | ACSL5 | na |  |  | 14462 | -0.439 | -0.0395 | No |
| 104 | ACADVL | na |  |  | 14586 | -0.460 | -0.0409 | No |
| 105 | HSD17B4 | na |  |  | 14809 | -0.499 | -0.0471 | No |
| 106 | ALDH3A1 | na |  |  | 14969 | -0.509 | -0.0499 | No |
| 107 | IL4I1 | na |  |  | 15015 | -0.513 | -0.0467 | No |
| 108 | VNN1 | na |  |  | 15302 | -0.564 | -0.0556 | No |
| 109 | TP53INP2 | na |  |  | 15727 | -0.632 | -0.0710 | No |
| 110 | EPHX1 | na |  |  | 15777 | -0.644 | -0.0665 | No |
| 111 | SERINC1 | na |  |  | 15845 | -0.656 | -0.0629 | No |
| 112 | GRHPR | na |  |  | 16141 | -0.715 | -0.0706 | No |
| 113 | NTHL1 | na |  |  | 16187 | -0.728 | -0.0651 | No |
| 114 | ECH1 | na |  |  | 16356 | -0.770 | -0.0655 | No |
| 115 | ACADS | na |  |  | 16402 | -0.782 | -0.0593 | No |
| 116 | ALDOA | na |  |  | 16455 | -0.799 | -0.0534 | No |
| 117 | UBE2L6 | na |  |  | 16481 | -0.806 | -0.0459 | No |
| 118 | HMGCL | na |  |  | 16532 | -0.819 | -0.0397 | No |
| 119 | GSTZ1 | na |  |  | 16561 | -0.826 | -0.0322 | No |
| 120 | LGALS1 | na |  |  | 16602 | -0.835 | -0.0252 | No |
| 121 | REEP6 | na |  |  | 16765 | -0.874 | -0.0242 | No |
| 122 | PSME1 | na |  |  | 16865 | -0.910 | -0.0195 | No |
| 123 | BPHL | na |  |  | 16938 | -0.932 | -0.0131 | No |
| 124 | CEL | na |  |  | 17224 | -1.014 | -0.0170 | No |
| 125 | GLUL | na |  |  | 17365 | -1.064 | -0.0128 | No |
| 126 | ACOT8 | na |  |  | 17936 | -1.295 | -0.0286 | No |
| 127 | S100A10 | na |  |  | 18028 | -1.345 | -0.0188 | No |
| 128 | ALAD | na |  |  | 18280 | -1.479 | -0.0159 | No |
| 129 | CRAT | na |  |  | 18281 | -1.480 | 0.0002 | No |
| 130 | ENO2 | na |  |  | 18286 | -1.481 | 0.0161 | No |
| 131 | GABARAPL1 | na |  |  | 19059 | -2.914 | 0.0073 | No |
Table: GSEA details [plain text format]

  

Fig 2: HALLMARK\_FATTY\_ACID\_METABOLISM      
 Blue-Pink O' Gram in the Space of the Analyzed GeneSet

  

Fig 3: HALLMARK\_FATTY\_ACID\_METABOLISM: Random ES distribution      
 Gene set null distribution of ES for **HALLMARK\_FATTY\_ACID\_METABOLISM**
